# Genetic and training adaptations in the Haenyeo divers of Jeju, Korea

**DOI:** 10.1016/j.celrep.2025.115577

**Published:** 2025-05-02

**Authors:** Diana Aguilar-Gómez, Jacob Bejder, Jonathan Graae, Yelin Ko, Andrew Vaughn, Kendell Clement, Martin Tristani-Firouzi, Joo-Young Lee, Nikolai B. Nordsborg, Rasmus Nielsen, Melissa Ilardo

**Affiliations:** 1 Ecology and Evolutionary Biology, University of California, Los Angeles, 612 Charles E. Young Drive South, Los Angeles, CA 90095, USA; 2 Center for Computational Biology, University of California, Berkeley, Berkeley, CA 94720, USA; 3 Department of Human-Centered Design, Cornell University, Ithaca, NY 14850, USA; 4 Department of Nutrition, Exercise, and Sports, University of Copenhagen, Nørre Allé 51, 2200 Copenhagen, Denmark; 5 Department of Biomedical Informatics, University of Utah, 421 Wakara Way #140, Salt Lake City, UT 84108, USA; 6 Division of Pediatric Cardiology, Department of Pediatrics, University of Utah, 81 Mario Capecchi Dr., Salt Lake City, UT, USA; 7 Nora Eccles Harrison CVRTI Cardiovascular Research Training Institute, University of Utah, Salt Lake City, UT, USA; 8 Research Institute for Human Ecology, College of Human Ecology, Seoul National University, Seoul 08826, Republic of Korea; 9 Graphene Research Center for Convergence Technology, Advanced Institute of Convergence Technology, Suwon 16229, Republic of Korea; 10 Lead contact

## Abstract

Natural selection and relative isolation have shaped the genetics and physiology of unique human populations from Greenland to Tibet. Another such population is the Haenyeo, the all-female Korean divers renowned for their remarkable diving abilities in frigid waters. Apnea diving induces considerable physiological strain, particularly in females diving throughout pregnancy. In this study, we explore the hypothesis that breath-hold diving has shaped physiological and genetic traits in the Haenyeo. We identified pronounced bradycardia during diving, a likely training effect. We paired natural selection and genetic association analyses to investigate adaptive genetic variation that may mitigate the effects of diving on pregnancy through an associated reduction of diastolic blood pressure. Finally, we identified positively selected variation in a gene previously associated with cold water tolerance, which may contribute to reduced hypothermia susceptibility. These findings highlight the importance of traditional diving populations for understanding genetic and physiological adaptation.

## In brief

Aguilar-Gómez et al. find evidence of evolutionary adaptation with potential relevance to breath-hold diving in the Haenyeo divers of Korea. Genetic variants under selection in this population may help mitigate the physiological strain of repeated diving during pregnancy. Additionally, long-term training appears to enhance the diving response through pronounced bradycardia.

## Graphical Abstract

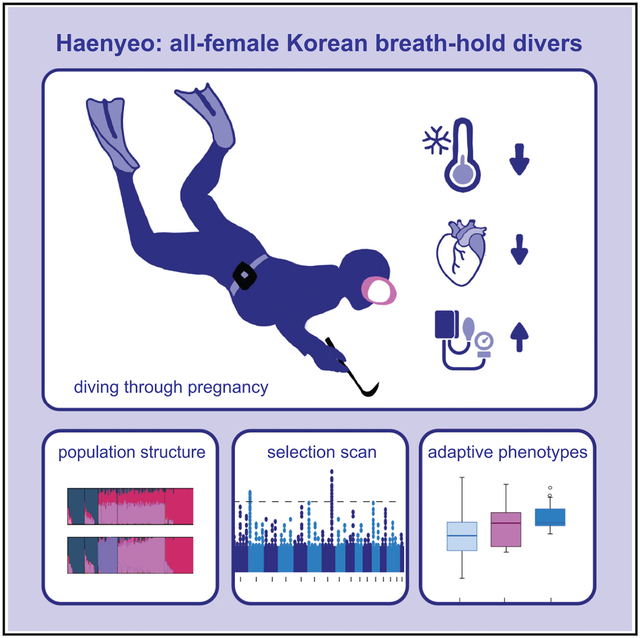

## INTRODUCTION

Extraordinary human populations have inhabited nearly every environment on the planet, adopting unique lifestyles and strategies to survive. The extreme conditions faced by these populations have imposed natural selection, introducing heritable adaptive variation. Modern humans have adapted to high altitudes,^1,2^ distinctive diets,^3^ and even breath-hold (or apnea) diving.^4,5^ Studying the genetics and physiology of these populations through the lens of evolution enables a deep understanding of the relationship between genetic variation and human physiology, including novel discoveries that cannot be found through genome-wide association studies (GWAS) alone. We present evidence of a novel adaptation in the second known population that may have evolved for breath-hold diving: the Haenyeo of Jeju Island, Korea.

The inhabitants of Jeju Island represent a subpopulation of Korea with a distinct language, a matriarchal family structure, and the celebrated resident community of Haenyeo divers. The practice of breath-hold diving is so integral to Jeju’s culture that the shortening of words characteristic of the Jeju language is colloquially attributed to the need for divers to communicate quickly at the water’s surface. Haenyeo literally translates to women of the sea.^6^ The Haenyeo dive year-round in social collectives, harvesting everything from abalone to sea urchin. Their typical diving occurs at relatively shallow depths (<10 m) and for a short duration (~30 s) but with high energy expenditure for around 4–5 h per day.^7^ Haenyeo typically dive throughout pregnancy, often continuing to dive until the day they give birth.^8^ Young women are no longer continuing this matrilineal tradition; the current Haenyeo population, with an average age of around 70 years, may represent the last generation of Haenyeo divers.^9^

During diving, all humans experience a well-characterized effect known as the mammalian dive reflex.^10–14^ This multi-system physiological response offers many biological components on which natural selection can act. The dive reflex is triggered by the combination of apnea and water submersion and induces vasoconstriction, which selectively redistributes blood to vital organs and away from non-essential tissues.^15,16^ Diving also triggers a slowing of the heart rate, or bradycardia, which reduces cardiac output and conserves oxygen.^10,17,18^ The only element of the dive response thus far demonstrated to have a link to natural selection is spleen size, which was found to be increased in the Bajau “sea nomads” of Indonesia. During a dive, splenic contraction increases the volume of circulating red blood cells and, thus, available oxygen by up to 9.6%.^19–22^ Other than spleen size, it is unknown to which extent components of the dive response are influenced by genetic variation rather than training. Genetic evidence from study of the Bajau and others suggests that variation in the gene *BDKRB2* may have been selected for its role in vascular contraction^5,23^; however, this effect has not yet been directly measured in any traditional diving population.

The interaction between the mammalian dive reflex and pregnancy poses unique physiological challenges for female divers. Although there is limited documentation of its impact in breath-hold divers, exposure to intermittent hypoxia during early pregnancy is linked to a higher risk of hypertensive disorders of pregnancy, commonly in the form of preeclampsia.^24^ This effect has most commonly been observed in cases of obstructive sleep apnea (OSA), where apnea triggers vasoconstriction, raising blood pressure.^25,26^ Given the parallels between OSA and breath-hold diving, it is plausible that apnea during diving may also increase preeclampsia risk.

The genetics of diving physiology in the Haenyeo and the broader Jeju population have not been studied to date. We were motivated by the discovery of natural selection on diving in the Bajau to pursue the first investigation of genetic adaptation to diving in the Haenyeo. Our participants included Haenyeo divers and age-matched controls in Jeju and Seoul. We measured baseline metrics associated with diving physiology, including spleen size. We also performed cardiovascular measurements at rest and during a series of simulated dives to identify candidate diving phenotypes that may be associated with underlying genetic variation. We then used whole genome sequencing (WGS) data to identify genetic loci that have been under selection in this population and examined whether these loci were associated with candidate phenotypes.

## RESULTS

### Study population

We sampled women from three populations: Haenyeo in Jeju (*n* = 30), non-Haenyeo in Jeju (*n* = 30), and non-Haenyeo in Seoul (*n* = 31). These sample sizes were selected to match those of prior studies that have detected genetic association on selected genetic variation in genetically isolated, putatively adapted populations.^1,2,5^ We chose to include participants from the Korean mainland to account for possible genetic continuity between the Haenyeo and non-Haenyeo participants on Jeju Island as well as possible genetic differences between Jeju and mainland Koreans. All Haenyeo participants in Jeju were self-identified members of a Haenyeo community in Hado, Jeju, and were all at least third-generation Haenyeo. All Haenyeo were actively working as divers, spending a minimum of 3 days per week in the water. Non-Haenyeo controls in Jeju and Seoul were all age matched to reflect the demographics of the Haenyeo, an aging population (average age at the time of the study, 65 years). Using genetic analyses, we identified three individuals as being closely related, two from Jeju and one from Seoul, and excluded these individuals from all subsequent analyses.

### Spleen measurements

Previous work demonstrated that naturally selected genetic variation was associated with an increased spleen size in a traditional diving population,^5^ and subsequent work has suggested that the act of apnea training itself can correlate with an increase in spleen size.^27^ We therefore measured spleen size in the Haenyeo to compare with control populations. We performed ultrasound measurements in two planes in order to calculate spleen volume using the same methodology employed in the Bajau^5^ that has been demonstrated to correlate well with computed tomography measurements. We found that spleen size did not differ between the Haenyeo and non-Haenyeo of Jeju; however, the spleen size of each Jeju group was significantly larger than that of controls from Seoul (two sample t test, *p* = 0.0075 and *p* = 0.016 for Haenyeo vs. Seoul and Jeju vs. Seoul, respectively). When using a linear model to test Jeju individuals compared to non-Jeju individuals, the results remained significant (*p* = 0.016, β = −85.42, SE = 34.75). However, when we accounted for additional covariates, including height, weight, and age, the results were no longer significant (*p* = 0.052, β = −88.59 SE = 44.96). This suggests that the observed spleen size difference may not be primarily correlated with genetic effects.

### Hematological measurements

The large spleen adaptation in the Bajau is linked to genetic variation that is associated with changes in thyroid hormone levels. Hyperthyroidism is known to stimulate erythropoiesis,^28^ and recent work in animal models suggests that replicating the Bajau genetic adaptation is associated with hematological variation.^29^ Specifically, mice that underwent pharmacological inhibition of the same gene thought to be downregulated in the Bajau presented with higher red blood cell (RBC) count, hemoglobin (Hgb), and hematocrit (Hct) than control animals. On the basis of these results, we used capillary blood samples to measure baseline hematological parameters in all participants. We found no difference in Hgb or Hct between any of the groups measured.

### Simulated dive measurements

We sought to determine whether natural selection on breath-hold diving is associated with changes in Haenyeo physiology. We conducted a series of simulated dives to measure diving-specific physiological responses through a protocol refined in previous studies of the human dive response.^23,30^ In this sequence, the participant maximally holds her breath while submerging her face in a pool of cold water, thus triggering the bradycardia and peripheral vasoconstriction associated with the human dive reflex. Over a series of three “dives,” with 2 min recovery between each dive, we measured the cardiovascular response to the diving stimulus in the form of heart rate and blood pressure (BP).

**Figure 1.**
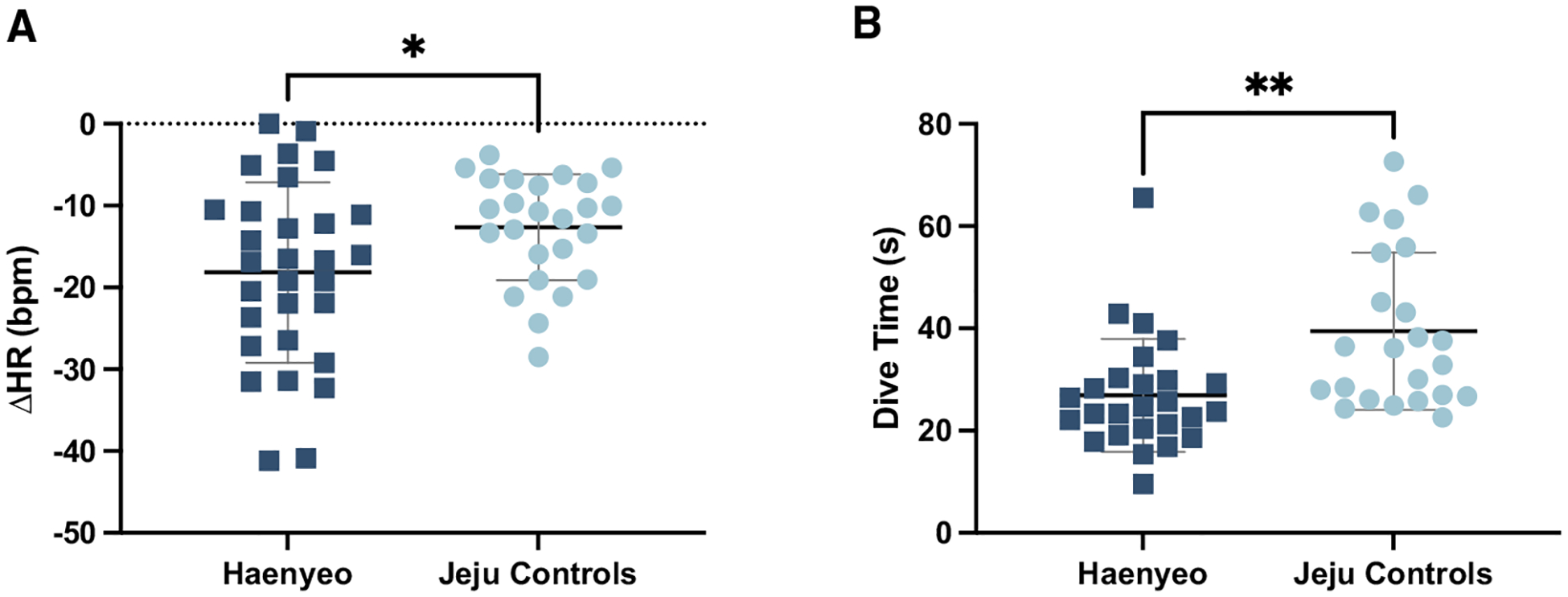
Haenyeo display a significant decrease in HR compared to Jeju controls during a simulated dive despite significantly shorter dive times (A and B) Due to the shared ancestry within the population of Jeju, this is likely a training effect rather than determined by genetic variation. Data are represented as mean ± SD. Statistical significance was determined using an unpaired t test. *p ≤ 0.05, **p ≤ 0.01.

We first quantified the extent of bradycardia over simulated dives. While participants from all groups experienced bradycardia during diving, we found that the Haenyeo demonstrated significantly greater bradycardia over the course of a simulated dive than either control group. The difference in bradycardia was particularly pronounced within Jeju populations; the Haenyeo displayed a mean difference in heart rate (ΔHR) of −18.8 bpm compared to −12.6 bpm in Jeju controls (*p* = 0.015) despite significantly shorter dive times of 27 s in the Haenyeo and 39 s in Jeju controls, respectively (*p* = 0.0018) (Figure 1).

**Figure 2.**
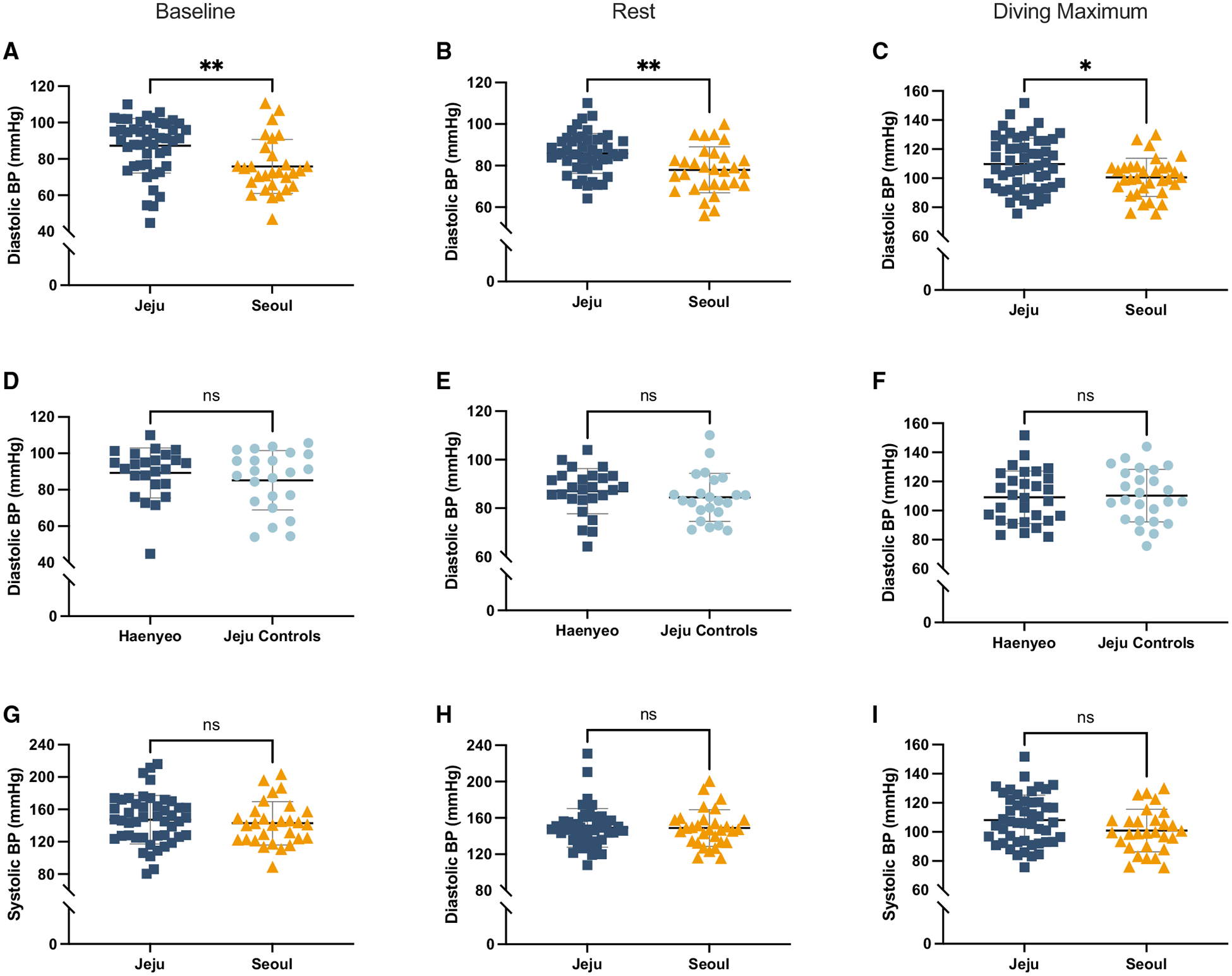
Diastolic BP is significantly higher in Jeju residents than in Seoul controls at all measured time points (A–C) Diastolic BP is higher in Jeju participants than in Seoul participants at baseline (A), during a simulated dive (B), and during the 2 min recovery between dives (C). (D–F) Diastolic BP is not significantly different between Jeju controls and Haenyeo at any of the same time points. These results suggest underlying genetic variation that contributes to elevated diastolic BP. (G–I) There is no difference in Systolic BP between Jeju and Seoul at any time point. Data are represented as mean ± SD. Statistical significance was determined using an unpaired t test. ns, not significant (*p* > 0.05); *p ≤ 0.05, **p ≤ 0.01.

We then examined diastolic and systolic BP. We considered these two measurements separately rather than mean arterial pressure (which is calculated as the sum of 2/3 diastolic BP and 1/3 systolic BP) in order to detect any independent changes in either variable that could be obscured by combining them. We found that the Haenyeo experience significantly higher diastolic BP on average than controls from Seoul at baseline (*p* = 0.010), during dives (*p* = 0.0053), and during the 2 min recovery period between dives (*p* = 0.00051) (Figures 2A–2C). However, diastolic BP was not significantly different between Haenyeo and Jeju controls. Isolated diastolic hypertension has not been observed previously in diving populations. The difference in diastolic BP was consistently approximately 10 mm Hg higher in Jeju residents than in Seoul controls (Figures 2D–2F). Systolic BP was not significantly different between any groups at any time point (Figures 2G–2I). Because we were most interested in natural selection on diving, we chose to focus on the measurement of diastolic BP that might best represent a physiological benefit during a dive through its effect on increasing cerebral perfusion: maximum diastolic BP reached over a simulated dive. Specifically, we examined the first simulated dive in the series. Selecting this measurement also enabled us to control for possible confounding factors through the inclusion of dive duration as a covariate. We found that Jeju residents, regardless of whether they are Haenyeo, have significantly higher maximum diastolic BP during the simulated dive (*p* = 0.014 using a two-sample t test). Using a multiple linear regression model to account for height, weight, age, and dive duration, the diastolic BP difference between groups remained significant (*p* = 0.012, β = −10.1, SE = 3.90). This finding is consistent with a previous survey that indicates that Jeju has one of the highest rates of hypertension in the nation.^31^

### Genetic analyses

#### Demographic and evolutionary history

To examine the demographic and evolutionary history of the Jeju population, we generated low-depth WGS data (5×), coverage selected deliberately to be used in conjunction with genome imputation.^32^ As a reference panel for imputation, we used Korean mainland individuals, specifically from the Korean Genome Project (Korean1K).^33^ Based on DNA sample quality, we were able to generate sequencing data from 84 individuals representing Haenyeo in Jeju (*n* = 27), non-Haenyeo in Jeju (*n* = 30), and non-Haenyeo in Seoul (*n* = 27).

**Figure 3.**
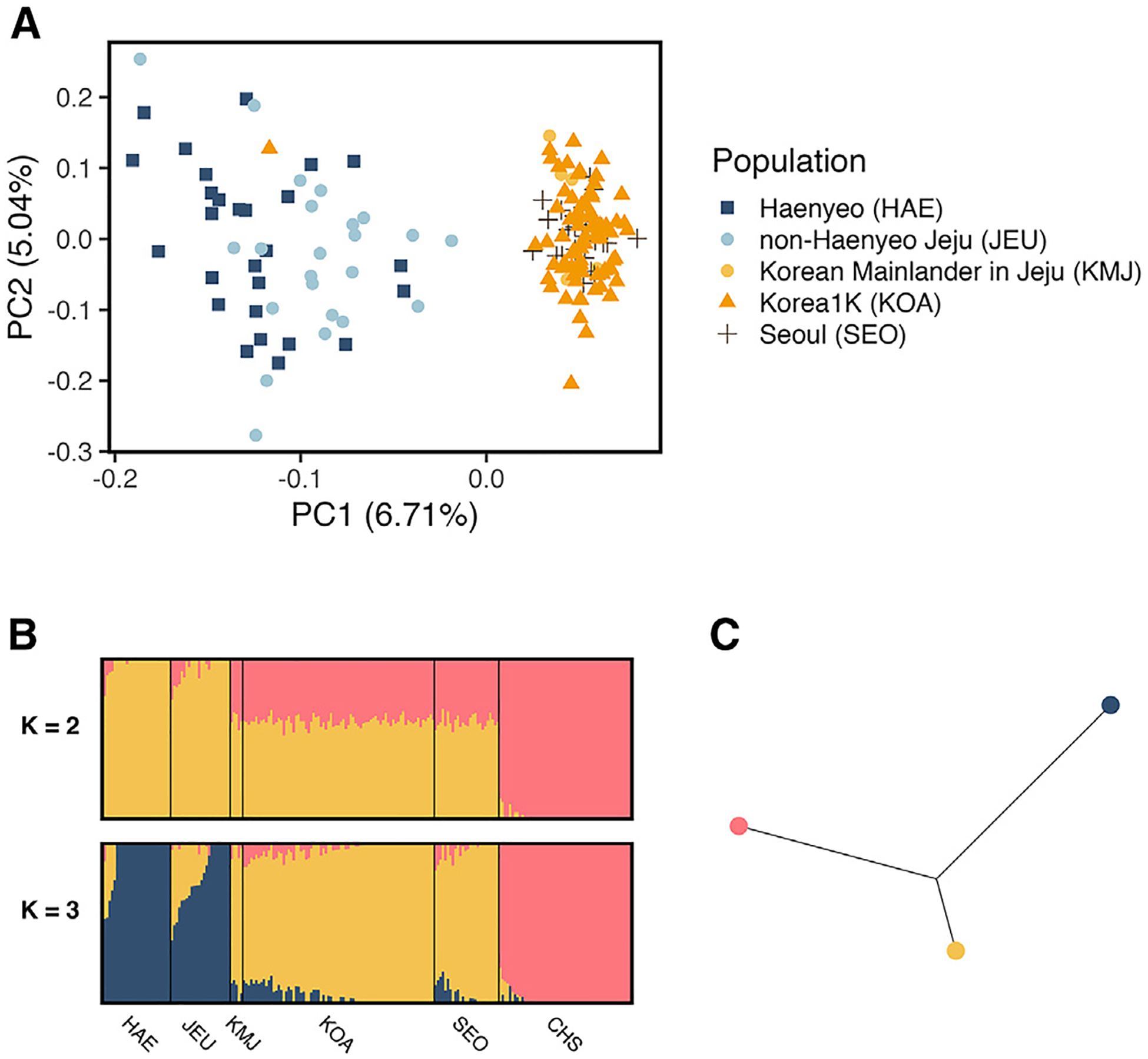
Genetic relationships between the Haenyeo, Jeju Island controls, and mainland Koreans (A) In a PCA of only Korean individuals, the Haenyeo cluster with other JEUs, and all Jeju residents separate from mainland Koreans. F_ST_ values indicate that the Jeju Island populations are approximately panmictic, sharing an ancestral population, and distinct from the Korean mainland populations. (B) Admixture graph at *K* = 2 and *K* = 3 for the Haenyeo, mainland Koreans, and Han Chinese highlights the genetic difference between Jeju individuals (Haenyeo Jeju controls) and mainland Koreans, with Jeju individuals receiving their own, distinct component. (C) Admixture tree estimate for the components in (B) at *K* = 3. The Jeju branch of the tree estimate is notably the longest of the three.

For the purpose of population history analyses, we merged the data we collected with data from the Korean1K data and selected populations from the 1000 Genomes Project dataset, resulting in 5,673,091 SNPs after merging and filtering. We chose to include only female participants for consistency with the individuals in our study. We performed a principal-component analysis (PCA) including only individuals from Korea: Haenyeo (HAE), non-Haenyeo Jeju residents (JEU), Korean mainlanders in Jeju (KMJ), participants from the Korean1K project (KOA), and controls from Seoul (SEO). In this PCA, PC1 appears to separate individuals from Jeju Island and those from the Korean mainland (Figure 3). The individuals in our dataset from Seoul cluster with those from Korean1K, who were primarily recruited in Ulsan on the Korean mainland.^33^ Five individuals from Jeju Island appeared to cluster tightly with mainland Koreans. In order to quantify this genetic similarity, we performed an admixture analysis using Ohana’s qpas^34^ and examined ancestry proportions of these individuals (Table S1). We relabeled any Jeju participants with more than 60% Korean ancestry and less than 15% Jeju ancestry as Korean mainlander in Jeju in subsequent analyses to more accurately reflect their genetic ancestry. Similarly, one individual from Korean1K clustered with the individuals from Jeju Island in the PCA. This individual had nearly 100% Jeju ancestry; therefore we reclassified this individual as a Jeju Islander (JEU).

The Haenyeo and Jeju controls appeared to be largely overlapping in PC1/PC2; however there was some apparent visual separation in the populations. We calculated the fixation index (F_ST_) to quantify the genetic divergence between these populations and found that the Haenyeo and Jeju controls are not meaningfully genetically differentiated (F_ST_ = 0.00031) (Table 1). This suggests that all individuals from multigenerational Jeju families, regardless of whether they are Haenyeo, represent an approximately panmictic population. Given the integral role of diving for thousands of years of Jeju’s history, it is therefore likely that the ancestors of all Jeju residents (Haenyeo and non-Haenyeo) are equally likely to be influenced by natural selection on diving. The ancestral separation between Jeju individuals and mainland Koreans is also reflected in F_ST_; the F_ST_ between Jeju Island and Mainland Korea is 0.0034. This suggests that mainland Koreans are at least as differentiated from Jeju residents than they are from the Japanese (JPT) (F_ST_ = 0.0030) and northern Han Chinese (CHB) (F_ST_ = 0.0026). We note, however, that F_ST_ reflects genetic divergence, which depends on effective population size (*N*_*e*_). The higher F_ST_ values may thus reflect a low *N*_*e*_ in the Jeju relative to CHB and JPT.

**Figure 4.**
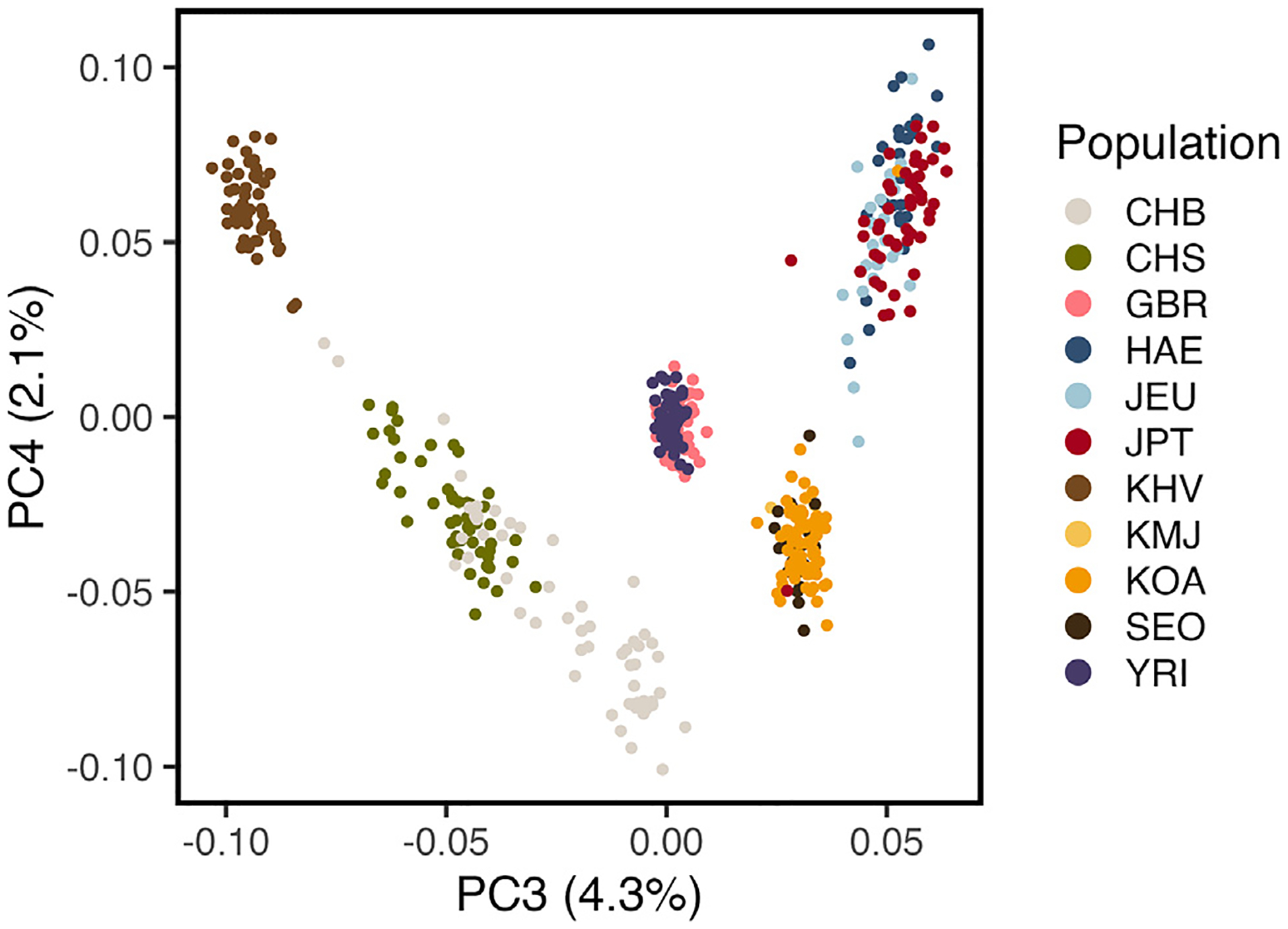
Global PCA of participants from this study as well as individuals from the 1000 Genomes Project and Korean1K In these PCs, residents of Jeju Island cluster with JPT individuals rather than with mainland Koreans.

**Figure 5.**
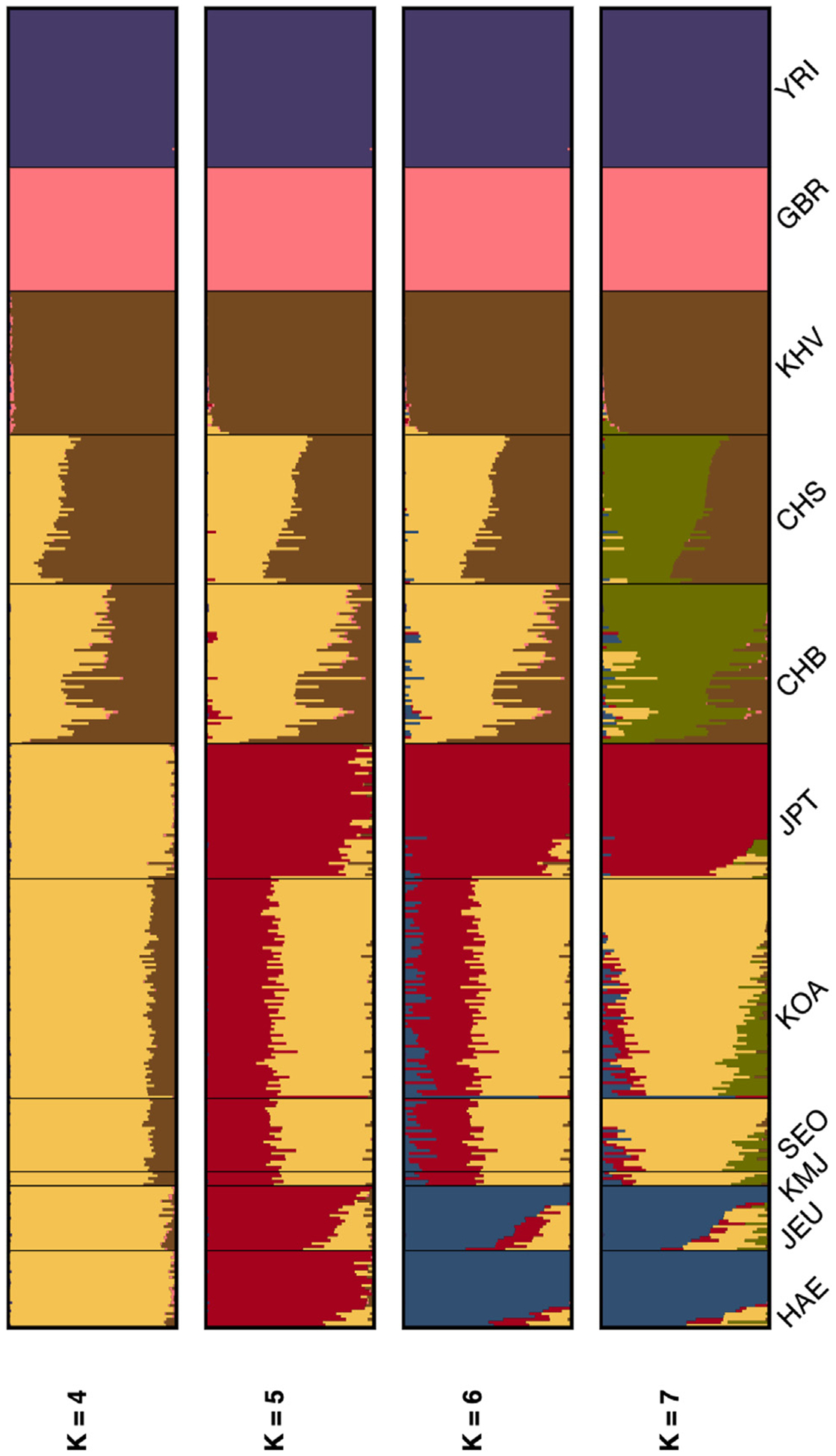
Admixture graph for the Haenyeo and other global populations The Haenyeo receive their own, distinct ancestral component at *K* = 6.

We performed a PCA using global populations from the 1000 Genomes Project, including four Asian populations and two outgroup populations. We observed that, in PC3 and PC4, the PCs in which Asian populations begin to separate from each other, individuals from Jeju cluster most closely with Japanese individuals rather than mainland Koreans (Figure 4). In additional admixture analyses of all populations using Ohana’s qpas,^34^ we found that, when considering 5 ancestry components (*K* = 5), two predominant ancestral components were represented in Northern East Asia: one shared by the Chinese and Mainland Koreans and a second specific to the Japanese and all Koreans (Figure 5). This component represents almost the complete ancestry of Japanese and Jeju individuals at this *K*, echoing the PC3/PC4 overlap between Jeju individuals and the JPT and reflecting shared ancestry between these populations. At *K* = 6, Jeju individuals are represented by their own ancestral component, and at *K* = 7, mainland Koreans are represented by another distinct component.

To contextualize any potential genetic adaptation, we examined the genetic history of Jeju. This history enables us to identify how and when adaptive variation may have arisen in the population. We started by using AdmixtureBayes to infer a population tree from the populations in our dataset. We ran AdmixtureBayes allowing for 0, 1, or 2 admixture events. When considering graphs with 0 admixture events, we saw that the Jeju Islanders (JEJ) and JPT populations were inferred to be sister populations, which is in concordance with the PCA showing significant overlap between the JEJ and JPT clusters (Figure S1A). The next most related population to both JEJ and JPT was inferred to be the mainland Koreans (KOR). When allowing for 1 admixture event, we inferred that the Southern Chinese population (CHS) is admixed between a population related to CHB and a population related to Kinh Vietnamese (KHV) (Figure S1B). These results are also in agreement with the PCA analysis showing the CHS cluster as being between the CHB and KHV clusters. The relationship between the JEJ, JPT, and KOR populations remained consistent with the 0 admixture event graph. There is some uncertainty in the exact placement of the CHB lineage as a result of the short branch connecting this population to the rest of the graph. When allowing for 2 admixture events, we found that the distribution of admixture graphs is too dispersed to draw definitive conclusions (Figure S1C). This is due to the very high number of admixture graph topologies with 2 admixture events that are compatible with the observed allele frequency covariance matrix. It is possible that using more information, such as identity by descent tracts, could help resolve more fine-scale admixture in these populations, but existing admixture graph inference methods are unable to do so.

We next wanted to estimate when the divergence between Jeju residents and mainland Koreans occurred as well as to put an upper limit on the time at which natural selection may have begun to shape Jeju genetic ancestry. We therefore estimated the *N*_*e*_ jointly for mainland Koreans and Jeju residents, from which we can then infer divergence time as the time when these populations stop sharing patterns of expansion or contraction. We found that the population trajectories of Jeju and mainland Koreans noticeably diverged roughly 5,000–7,000 years ago (Figure S2). Since this time, many other East Asian populations have experienced uninterrupted growth, resulting in a roughly 10-fold increase in *N*_*e*_ over the past 10,000 years. However, Jeju’s population has only effectively doubled in the past 10,000 years, with a dramatic reduction in population roughly 1,200–3,000 years ago, suggesting a bottleneck. We note that inferring divergence times from *N*_*e*_ curves involves assumptions about the demographic history of populations, such as absence of migration.

#### Selection analysis and association testing

To identify genes or variants that may be of adaptive significance and related to the phenotypes of interest, we used a two-step approach that previously has proven useful for detecting human adaptive genetic variation.^2,3,5^ We first scanned the genome for variants that may have been targeted by selection by comparing allele frequencies between populations. We subsequently tested for associations only on the top candidates from the selection scan. While only applicable in settings where there are closely related populations that differ phenotypically, this protocol greatly reduces the multiple testing burden of GWASs by focusing only on variants that have the potential to explain between-population phenotypic differences. This protocol has been used previously to detect variants in the *EPAS1* gene associated with altitude adaptation in Tibetans,^2^ variation in *FADS* genes associated with multiple phenotypes in Inuit,^3^ and genetic variants associated with diving-related phenotypes in the Bajau.^5^

**Figure 6.**
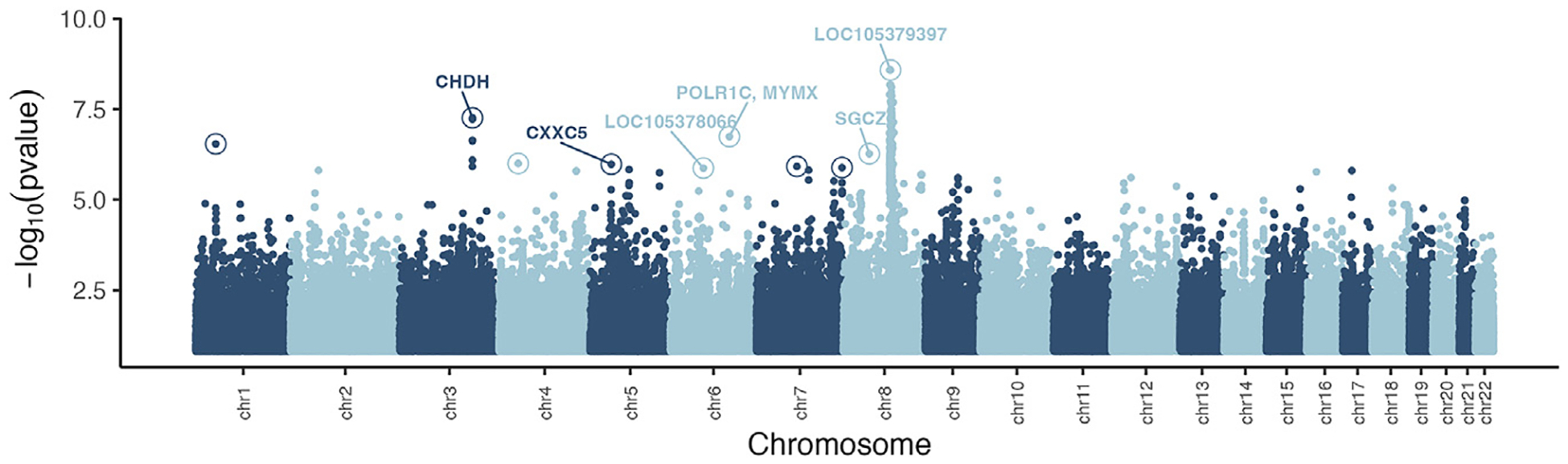
Manhattan plot of selection scan results, with the top 10 SNPs found in genomic regions under selection circled SNPs are further annotated with gene name if the SNP was found within a gene.

Our population genomics analyses on structure and demography revealed that the modern population of Jeju, including Haenyeo and non-Haenyeo, is descended from the same ancestral population. This indicates that modern Jeju residents are equally likely to have descended from ancestral divers, suggesting that a selection scan targeting the Jeju ancestral component is appropriate for detecting regions of positive selection in the Haenyeo and other Jeju residents. In order to detect naturally selected genetic variation, we used a method that leverages local patterns of allele frequency differences, similar to those used in previous selection studies.^2,3,5^ Specifically, we used selscan from Ohana^34^ to identify SNPs that deviate in Jeju from the genome-wide covariance using a likelihood ratio test. This statistical test identifies variants that have experienced a stronger change in allele frequency change between populations than expected given the genome-wide patterns of allele frequency change, and it assumes a very simple Brownian motion model of allele frequency change. We therefore also later apply a more parametric test (CLUES2^35^) that models selection and genetic drift using a Wright-Fisher model under an explicit demographic model that includes changes in population size in order to validate the selection inferences for the top candidate SNPs (this validation step is described in detail below). For the Ohana analyses, we selected mainland Koreans (KOR) as a comparison population and CHS as an outgroup. We elected to filter these results in order to annotate and further investigate the 10 strongest candidate signals of selection (Figure 6; Table S2). Among these results, we identified several SNPs in genetic regions with potential links to Haenyeo diving.

The strongest selection signal came from a peak in the region of the centromere of chromosome 8, upstream of the gene that encodes prostate, ovary, testis, and placenta expressed (POTE) ankyrin domain family member A. This peak overlaps with a signal previously identified through a GWAS in a Korean cohort, specifically a study of copy number variations associated with hematological parameters.^36^ Genetic variation in the region of our top SNP was significantly associated with RBC count. Hematological variation is believed to underlie the large-spleen phenotype observed in the Bajau and may provide additional oxygen capacity during diving.^29^ We observed another selected allele in the gene that encodes sarcoglycan zeta. Variation in this gene has been shown previously to be significantly associated with pain sensitivity, as measured by the cold pressor test in a 23andMe cohort of undisclosed (likely European) ancestry.^37^ This test, much like Haenyeo diving in the winter, involves submersion in exceptionally cold water. It may therefore be linked to the well documented Haenyeo ability to tolerate cold.^38–43^ While we did not measure thermoregulatory physiology in our study, this represents an avenue for future research.

We next wanted to test for an association between the variants putatively under selection in Jeju and diving diastolic BP. Using all individuals, including those from Jeju and Seoul, we used PLINK^44^ on the top SNPs from the 10 highest-scoring genomic regions in the selection scan to measure association with untransformed maximum diastolic BP during the first simulated dive. We included the following covariates: age, height, weight, dive length, and the first 10 PCs (to control for underlying population structure). We found that only one SNP from the selection scan was significantly associated with maximum diving diastolic BP at the Bonferroni-corrected significance level (0.005) using an additive model: rs66930627 (*p* = 0.0021). When we included age^1^ as an additional covariate to account for non-linear effects, as is common in BP GWASs,^45^ the association remained significant (*p* = 0.0014). We also tested the association within only Jeju participants to assess whether the association reflects population-specific allele frequency differences between Jeju and Seoul and found that the association remains significant (*p* = 0.0039). We note that we were unable to include use of anti-hypertensives as an additional covariate given that these data were not collected at the time of the study.

In order to validate the evidence for selection on this variant, we used an entirely independent method of detecting selection that accounts for changes in population size and demography and uses information from the haplotype and allele frequency distribution within the focal population: CLUES2.^35^ In contrast to the previous test, this method does not use allele frequency differences between populations but, instead, independent evidence of allele frequency changes obtained from the ancestral recombination graph. Using CLUES2, we found significant evidence in rs66930627 of selection favoring the C allele in the JEJ population across all 3 models considered: a 1-epoch model in which selection was constant through time (*p* = 3.3 × 10^−5^), a 2-epoch model in which the selection coefficient changed 1,200 years ago (*p* = 5.6 × 10^−5^), and a 2-epoch model in which the selection coefficient changed 7,000 years ago (*p* = 1.0 × 10^−4^). As measured by Akaike information criterion, an estimator of prediction error, the 2-epoch model with selective pressure beginning 1,200 years ago was chosen as the best model, thus providing evidence in support of the hypothesis that the selection on rs66930627 to decrease diastolic BP was very recent and very strong (${\hat{S}}^{MLE}=0.021$ in the more recent epoch) (Figure S3). Interestingly, this timing aligns with a recent bottleneck experienced by the Jeju population.

The putatively selected allele in Jeju, C, has an allele frequency of 33% in Jeju and 7% in mainland Korea. It is associated with a decrease in diastolic BP corresponding to roughly a 10 mm Hg decrease in diastolic BP per selected allele. The established contribution of elevated diastolic BP to preeclampsia risk in pregnancy could induce a strong selective pressure, especially when provoked through the dive response. The association between rs66930627 and a decreased diastolic BP could therefore suggest that natural selection acted to mitigate elevated diastolic BP in the Jeju population. The associated SNP is found in an intergenic region; however, it is an expression quantitative trait locus (eQTL) for a gene that encodes Fcγ receptor IIA (FcγRIIA, *p* = 1.2 × 10^−4^) (data source: GTEx Analysis Release v.8; dbGaP: phs000424.v8.p2). FcγRIIA is a low-affinity Fcγ receptor that binds IgG2 and may influence hypertension through its effect on the pro-inflammatory activities of vascular smooth muscle cells.^46,47^ Manipulation of the gene FcγRIIB, which encodes the largely homologous Fcγ receptor IIB, has been demonstrated to directly correlate with hypertension in animal models.^48^ In humans, variation in FcγRIIB is significantly associated with preeclampsia risk.^49^

The SNP rs66930627 is also an eQTL for FC receptor-like B (FCRLB; *p* = 7.0 × 10^−5^), which shares a common ancestor with FcγRIIB,^50^ and heat shock 70 kDa protein 7 (HSPA7; *p* = 1.2 × 10^−6^). HSPA7 is also referred to as a pseudogene or long non-coding RNA and has been shown to promote the inflammatory transition of vascular smooth muscle cells. HSPA7, like FCγRIIA and FCRLB, may therefore also play a role in inflammation-mediated hypertension.^51^ The functional overlap between the eQTLs of the associated SNP suggests a potential redundancy in their roles.

We were able to validate the association between rs66930627 and diastolic BP in a European ancestry cohort. In a sample of European ancestry individuals from the All of Us cohort (*n* = 1,376), filtered to age match our Korean cohort and exclude individuals on anti-hypertensives, we found a significant association between rs66930627 and diastolic BP (*p* = 0.040). In this cohort, we found that each additional C allele corresponded to a roughly 0.75 mm Hg decrease in diastolic BP, a more modest effect size than what we observed in the Korean cohort. This discrepancy underscores the challenges of validating such associations in populations with different genetic backgrounds.

## DISCUSSION

We present the first evidence of naturally selected genetic variation in the Haenyeo divers of Jeju Island, Korea. Using natural selection combined with association, we found a genetic variant that is significantly associated with diastolic BP. We also identified a training effect in the Haenyeo that likely contributes to their skillful diving. Populations like the Haenyeo are integral for uncovering relationships between genes and the regulation of physiology due to their demographic and evolutionary history.

Our first finding was that Jeju represents a genetically distinct subset of the Korean population. Jeju, which is found ~80 km off the Korean peninsula, has long been relatively isolated from the Korean mainland. The earliest signs of human settlement on Jeju date back 10,000 years to a community that eventually grew into the Tamna Kingdom. For a brief period roughly spanning the 14^th^ century, Mongolian forces controlled the island, possibly introducing genetic ancestry distinctive to the region.^52^ Mongol control was succeeded by half a millennium of Jeoson rule, during which Jeju faced significant restrictions with a lasting cultural impact. For example, inhabitants of Jeju were prohibited from leaving the island, thus increasing Jeju’s relative isolation until well into the 19^th^ century.^6^ Owing to this unique history, the Jeju language is by some measures as different from standard Korean as Dutch is from Norwegian.^53^ We found that this linguistic distinction is reflected in genetics; the Jeju population appears to be genetically diverged from mainland Korea. We also found that the population of Jeju is approximately panmictic, with Haenyeo divers and non-Haenyeo Jeju residents descending from the same ancestral population. For the purposes of distinguishing between genetic adaptation and training effects, Jeju residents can therefore be considered non-diving Haenyeo.

We found that the Haenyeo and other Jeju residents demonstrate significantly elevated diastolic BP compared to residents of Seoul at baseline, over the course of a simulated dive, and during recovery between dives. We found no corresponding elevation of systolic BP, although hypertension has been reported previously in Jeju.^31^ Elevated diastolic BP, particularly when exacerbated during apnea, has been linked to pregnancy risk factors, including preeclampsia.^54^ We identified a genetic variant with evidence of strong, recent natural selection in Jeju that is significantly associated with a reduction of diastolic BP in this population. This association may reflect natural selection to mitigate the complications of diastolic hypertension experienced by female divers while diving through pregnancy. Pregnancy is increasingly thought to be a driver of natural selection in extreme environments and lifestyles, with particular focus on the relationship between natural selection and pregnancy in women living at high altitude.^55^ Preeclampsia has a well-established relationship with long-term cardiovascular health outcomes.^56,57^ The BP mitigation correlated with the identified genetic variation may therefore also be linked to the observation that, while Jeju has the second highest prevalence of hypertension in Korea, it also has the lowest stroke mortality in the nation.^31^ Further data are needed to fully control for environmental factors that may influence regional differences in BP.

The genetic variant presumed to be under selection and associated with a decrease in diastolic BP is found in a region that is associated with changes in expression of the gene FcγRIIA. By binding IgG2, FcγRIIA may mitigate diastolic hypertension through its role in vascular inflammation, resembling the mechanism of the related Fcγ Receptor FcγRIIB.^46–48^ In fact, genetic variation in FcγRIIB is significantly associated with preeclampsia.^49^ This association may be linked to the mitigation of vascular inflammation. The nature of this relationship is unclear with current data, especially as our understanding of the role of inflammation in vascular biology and secondary hypertension continues to evolve.

We were able to validate the association between rs66930627 and diastolic BP in a European ancestry subset of the All of Us cohort, albeit with a lower-magnitude effect (~0.75 mm Hg). In the general population, a 1 mm Hg rise in systolic BP corresponds to a log-linear incremental risk of cardiovascular disease, so the effect size we observed in All of Us is likely clinically significant.^58^ BP is known to be a multifactorial and polygenic trait,^45^ and SNPs identified in GWASs have yet to provide sufficient magnitude effect to cause observed BP phenotypes outside of monogenic BP syndromes.^59^ Additionally, the majority of genetic variants previously identified as associated with BP traits are common rather than rare variants due to the late onset nature of hypertension, which protects this variation from purifying selection.^59,60^

We observed that the Haenyeo exhibit pronounced bradycardia in response to simulated diving, displaying a significantly greater reduction in HR over the course of a dive. Remarkably, one Haenyeo participant experienced an HR drop of over 40 bpm in a simulated dive lasting just 15 s. This feature appears to be a training effect, as it was only observed in diving Haenyeo and not in their non-diving genetic relatives in Jeju. These results are consistent with previous studies in the Haenyeo and in other trained divers, where rigorous apnea dive training has been suggested to increase the magnitude of bradycardia over a dive.^38,61–64^

We were able to demonstrate physiological outcomes in the Haenyeo divers of Jeju that may arise from both genetic adaptations and a lifetime of dedicated training. The Haenyeo are the second known breath-hold diving population in whom medically relevant physiology is correlated with novel, adaptive genetic variation. Our findings reinforce the extraordinary biology of diving communities and the power of natural selection to illuminate novel genetic variation. Continued investigation of populations like the Haenyeo will provide opportunities for breakthroughs in our understanding of the genetic regulation of disease.

### Limitations of the study

While our study illustrates the strength of leveraging an evolutionary approach to identify novel genetic variation associated with changes in physiology, it is limited by the sample size, the limited physiological data collected, and sequencing depth due to the constraints of the population and study magnitude. Generalizability of the results is limited by the fact that all participants were female. As with all studies of genetic adaptation in modern human populations, it is impossible to ever know the true selective pressure that is driving observed signals of selection, in part due to the pleiotropic nature of most genes. Furthermore, any parametric claims about selection in humans or other organisms using population genetic data rely on assumptions regarding population history and demography. Some uncertainty regarding the possible confounding effects of demography will always remain. A future study with more extensive experimental physiological, clinical, and environmental data will be necessary to more deeply unravel the genetic basis of diving adaptation and to better understand potential relationships with hypertensive disorders of pregnancy.

## RESOURCE AVAILABILITY

### Lead contact

Requests for further information should be directed to and will be fulfilled by the lead contact, Melissa Ilardo (melissa.ilardo@utah.edu).

### Materials availability

This study did not generate new unique reagents.

### Data and code availability

- Newly generated, de-identified WGS data have been deposited at the NCBI Sequence Read Archive and are publicly available as of the date of publication. Accession numbers are listed in the key resources table.
- All original code has been deposited at https://github.com/aguilar-gomez/haenyeo and is publicly available as of the date of publication.
- Any additional information required to reanalyze the data reported in this paper is available from the lead contact upon request.

## STAR★METHODS

### EXPERIMENTAL MODEL AND STUDY PARTICIPANT DETAILS

#### Human subjects research

We obtained approval to conduct these human subject studies from the IRB boards of both the University of Utah and Seoul National University. Prior to implementing the study, we conducted community engagement and outreach to gauge community interest in the project. Informed consent was obtained from all participants. All participants were consented by a native Korean speaker (who also spoke Jeju dialect) after a thorough explanation of the experimental protocol. Following data analysis, we returned to share the results with the communities to ensure comfort with the results and their presentation.

We recruited female participants from three groups: Haenyeo in Jeju (*n* = 30 indviduals), non-Haenyeo in Jeju (*n* = 30 individuals), and non-Haenyeo in Seoul (*n* = 31 individuals). All Haenyeo participants in Jeju were self-identified members of a Haenyeo community in Hado, Jeju and were all at least third-generation Haenyeo. All Haenyeo were actively working as divers, spending a minimum of 3 day per week in the water. Non-Haenyeo controls in Jeju and Seoul had no Haenyeo relatives and were all age matched to reflect the demographics of the Haenyeo, an aging population. Participants were all female due to the nature of the Haenyeo tradition. Participants had an average age of 65 years old. Participant selection is further described in the results section of the manuscript.

### METHOD DETAILS

We obtained and electronically recoded the following basic demographic information: age, self-identified ancestry, whether or not the individual is a diver, and how frequently they dive. We then measured the participants height and weight, took capillary blood samples, and measured spleen size at rest.

We next had each participant perform the diving protocol. We asked each participant perform a series of simulated dives through a standardized procedure that has been used in previous studies of the human dive response.^23^ In this procedure, the volunteer was asked to lie in a prone position on a comfortable, elevated surface. We attached a Nexfin monitor to the participant’s right hand middle finger to measure heart rate as well as systolic and diastolic blood pressure. After attaching the device, the participant performed a test ‘dive’ in order to acclimatize to the procedure, followed 10 minutes of rest. A ‘dive’ included the following: the volunteer took a deep breath and placed their face in a bowl of water that was 10°C cooler than room temperature and was elevated to the same height as the surface on which they were resting. When the volunteer could no longer hold their breath with reasonable comfort, they raised their face from the water and breathed normally. Following the test dive, the participant rested for an additional 10 minutes to return to a physiological baseline. The participant then performed three (non-test) dives with two minutes of rest between each dive.

### QUANTIFICATION AND STATISTICAL ANALYSIS

#### Physiological analyses

We tested between-group differences in heart rate, spleen volume, and blood pressure measurements using an unpaired t-test. For heart rate, our sample sizes were as follows: Jeju controls: *n* = 26, Jeju Haenyeo: *n* = 29, Seoul controls: *n* = 31. For spleen volume, our sample sizes were as follows: Jeju controls: *n* = 26, Jeju Haenyeo: *n* = 29, Seoul controls: *n* = 31. For all blood pressure measurements, our sample sizes were as follows: Jeju controls: n = 25, Jeju Haenyeo: n = 29, Seoul controls: n = 31. In Figures 1 and 2, we plotted the distribution of values with error bars where the center represents the mean and the error bars indicate one standard deviation. Statistical significance in the unpaired t test, as plotted, was defined as *ns* = not significant (*p* > 0.05), * = *p* ≤ 0.05, and ** = *p* ≤ 0.01 (See Figures 1 and 2).

The measurement we selected for genetic association testing represented the maximum diastolic blood pressure during the first (non-test) dive. This dive represented the dive in which the participants were maximally normalized to each other because of the preceding 10 minute rest period and because they had already experienced a test dive to acclimatize them to the experimental setup. To further interrogate diastolic blood pressure during diving, we used a multiple linear regression model to account for covariates that could affect this measurement. We included dive duration as a covariate in our multiple linear regression model to account for any differences arising from dive time. Further details on these analyses can be found in RESULTS, Simulated dive measurements.

#### Genetic analyses

##### Preprocessing reads

We first assessed the quality of the reads using FastQC v0.11.8, which generates summary statistics and visualizations to evaluate and identify potential biases or artifacts in the sequencing data.^66^ We found G repeats as overrepresented sequences and k-mer content. We did not detect adapter content; the base and sequence qualities were high with no warnings. We cleaned reads using PRINSEQ-lite 0.20.4, which filters and trims sequences based on quality scores, removes duplicate and low-complexity sequences, and eliminates reads containing ambiguous bases (Ns) to improve downstream analysis reliability.^67^ We filtered low-complexity reads using the dust method with a threshold of 7. We also included a custom parameter to delete repeats of 50 Gs in a row.

We mapped the reads to the human reference genome GRCh38 using *bwa-mem*,^68^ a Burrows-Wheeler Aligner algorithm optimized for high-throughput sequencing data. It performs local alignment, automatically detects split reads indicative of structural variants, and efficiently handles mismatches and indels, producing SAM format output for further processing. We used samtools^69^ to filter the bam files removing: unmapped reads, with mate unmapped, not primary alignment, reads that fail quality checks, and PCR or optical duplicates (-F 1804).

##### Imputation

Our average sequencing depth of 5x was selected deliberately for cost-effectiveness in a design based on imputation-based genotype calling. As a reference panel for the imputation, we used individuals from the Korean Genome Project (Korean1K).^33^ From this resource, we obtained a vcf file with data from 400 randomly chosen individuals. We refer to this dataset as Korean1K (KOA). We performed imputation using *GLIMPSE v1.1.1*.^32^ Using *bcftools view*,^70^ we only kept biallelic SNPs from the reference panel. We then calculated genotype likelihoods for each individual at each position. We did this by first using *bcftools mpileup* to annotate the number of reads in each position (-a ‘FORMAT/DP’), excluding indels (-I) and recalculating BAQ (-E), and then by *bcftools call* to calculate the likelihoods, using multiallelic caller (-m), inserting sited missed in *mpileup* but present in the reference panel (-i), and keeping all possible alternative alleles (-a). We next split the genome into pieces with a window size of 2Mb and buffer size of 200,000 (GLIMPSE_chunks). Then, we imputed each chromosome per individual (GLIMPSE_phase) by 2Mb piece. We ligated all pieces together (GLIMPSE_ligate) and phased the positions by sampling haplotypes (GLIMPSE_sample). Finally, we merged our files to generate a file for each chromosome with all the individuals using *bcftools merge*.

##### Relatedness

We calculated relatedness using ngsToolsV2,^71^ a revision of ngsRelateV1 that now allows for estimation of relatedness from low depth sequencing data even in the presence of endogamy. This program uses identity by descent (IBD) to quantify the genetic relationship between two individuals. K0 is the proportion of the genome where two individuals do not share any segments by IBD, and we therefore used 0.75 as a threshold for K0 to determine relatedness between any given pair of samples. We found five pairs of individuals with K0>0.75. The 10 individuals of these pairs were non-overlapping, so we excluded one individual from each pair and recalculated relatedness. The excluded individuals included two from Jeju, two from the Korea1K project and one from Seoul. We recalculated the relatedness for the individuals and no remaining pairs had K0>0.75.

##### Processing samples and SNP filtering

We downloaded data from individuals representing select global populations of the 1000 Genomes Phase 3 Project.^65^ All participants that make up our study data are female to match the demographics of the Haenyeo population. Therefore, we selected only female individuals from the 1000 genomes project for consistency. These individuals represented several East Asian populations; Japanese in Tokyo (JPT), Han Chinese In Beijing (CHB), Han Chinese South (CHS), and Kinh Vietnamese (KHV); as well as participants from Europe and Africa as outgroups: British in England and Scotland (GBR) and Yoruba from Nigeria (YRI).

We applied the strict 1000 genomes accessibility mask. We also filtered for biallelic sites, excluded sites with the flag ‘MAF=0’ to remove invariant sites, removed positions with the flag that the reference allele had been switched (‘INFO=REF_SWITCH’), and applied an excess of heterozygosity filter with *bcftools* (ExcHet<=.000001). These filters resulted in a set of 25,899,170 SNPs. We then merged the Korean data with the downloaded 1000 genomes samples and kept only positions present in both datasets, which resulted in a further reduction to 7,955,800 SNPs. We applied a 1% minor allele frequencies filter across individuals from all populations (*n* = 468 samples), resulting in 5,673,091 SNPs that were used in the downstream analyses.

##### Population structure analysis

We performed Principal Component Analysis (PCA) using plink,^44^ which computes eigenvectors and eigenvalues from genotype data to capture population structure and genetic variation. First, we performed a PCA with only Korean individuals. PC1 separated Jeju Island from the mainland. The individuals from Seoul in our dataset cluster with those from the Korean 1K project. One individual from the Korean 1K project clustered with the Jeju residents. After quantifying this individual’s ancestry and finding it consistent with Jeju, we re-labeled this individual as non-Haenyeo Jeju (JEU). We also identified five individuals from Jeju that appear to have predominantly mainland heritage, and we labeled them as Korean Mainlander in Jeju (KMJ). The remaining PCs do not further separate the Korean populations.

We also performed a PCA using all the Korean samples and other outgroup populations from the 1000 Genomes Project. We downloaded only female individuals from the following populations: Japanese in Tokyo (JPT), Han Chinese In Beijing (CHB), Han Chinese South (CHS), Kinh Vietnamese (KVH), British in England and Scotland (GBR) and Yoruba from Nigeria (YRI). PC1 and PC2 separate populations by continent. We observed three clusters: British (GBR), Yoruba (YRI), and Asian samples (HAE, JEU, SEO, KOA, JPT, CHB, CHS, KHV). PC3 and PC4 separate the Asian populations: PC3 separates populations in latitude, and PC4 separates them longitudinally. While Jeju Island individuals cluster with the Japanese in PC3/PC4, PC5 separates Japanese from Jeju Island individuals.

In order to estimate admixture proportions, we sampled 3% of sites to use as input for Ohana.^34^ We ran *qpas*, which estimates individual’s ancestry proportions assuming *k* ancestral components using maximum likelihood. We varied *k* from 4 to 7, with a maximum of 450 iterations, and used 0.08 as the minimum likelihood delta between iterations. From both the PCA and the structure analysis it became apparent that five of the individuals from Jeju (individuals 39,44,47,48, and 60) clustered and shared ancestry components with mainland Korean participants and therefore were relabeled as Korean mainlanders in Jeju (KMJ). At *k*=7, we noticed that one of the outgroups (YRI) was split into two groups, and we consequently used a supervised version of the algorithm, where individuals from the outgroups (YRI and GBR) were forced to have 100% ancestry from their respective outgroups. We used pong to visualize the structure plots.^78^ We used nemeco from Ohana to model the allele frequencies across ancestral components and obtain a matrix of the variances and covariances between them. We then used this matrix to estimate a tree of the ancestry components.

##### Demographic history and population divergence

In order to understand population divergence in our data, we calculated the Weir and Cockerman fixation index (F_ST_) using plink2 (–fst “CATPHENO” ‘method=wc’).^72,73^ F_ST_ values representing genetic differentiation between pairs of relevant populations can be found in Table 1.

We used two different methods for inferring a population tree and admixture events between populations: Treemix,^74^ which uses a Gaussian approximation for allele frequency change and a greedy search, and requires the user to specify the number of admixture events, and Admixture Bayes,^75^ which assumes a similar model but uses Bayesian inference and Markov Chain Monte Carlo to explore the space of possible admixture graphs. The latter method infers a credible set of admixture graphs and the number of admixture events from the data using a prior that penalizes having too many admixture events. For these analyses, we merged the individuals from Seoul and the Korean1K project into a single group labeled KOR. Similarly, we merged the Haenyeo and Jeju controls into a single population labeled JEJ and representing all Jeju residents. In both analyses, we used British individuals (GBR) as an outgroup. The graphs generated by both programs are consistent with each other and with our F_ST_, PCA, and admixture analyses.

To understand the effective population size (*N*_*e*_) history of Korean populations, we used the method implemented in Relate^76,77^ for estimating trajectories of *N*_*e*_. To provide an initial estimate of *N*_*e*_ for Ancestral recombination Graph (ARG) estimation in Relate, we first calculated the average number of pairwise differences (π) in KOR and JEJ populations using vcftools –windows-pi, setting the window size to 250,000,000, to get an estimate of π per chromosome. Then, we estimated effective population size using $\hat{N_{e}}=\pi /4\mu$. Using a mutation rate, μ=1.25e-8, the average *N*_*e*_ per chromosome was *N*_*eJEJ*_= 5204, and *N*_*eKOR*_=5194. We then estimated the ARG using Relate.^76,77^ We used *N*_*e*_= 5,000 as an initial estimate of population size in Relate to jointly infer population size over time for the JEJ and KOR populations. We chose a generation time of 27 years, consistent with previous estimates of generation times in East Asian populations^79^ We used Relate^76,77^ to infer ancestral recombination graphs (ARGs). We set the mutation rate to 1.25×10^−8^ and the effective population size of haplotypes (N) to 10,000. We then estimated population size over time for both the JEJ and KOR populations.

##### Selection scan

We performed a selection scan in order to identify regions of the Jeju population genome that have been under selection. We hypothesized that the dangers of breath hold diving, especially during pregnancy, combined with the integral history of this practice in Jeju, will have affected genetic variation in the population in ways that we can identify using selection analyses. For these analyses, we used the same 5,673,091 SNPs as those used in the demographic analyses. We focused on participants from only the following populations: Haenyeo (HAE), non-Haenyeo Jeju residents (JEU), Korean Mainlanders in Jeju (KMJ), Korean1K (KOA), Seoul (SEO), and Han Chinese South (CHS). Based on the structure analysis, two distinct Korean ancestral components emerged, one for HAE and JEU (representing Jeju heritage) and another for KOA, SEO, and KMJ (representing mainland heritage). We therefore decided to categorize the populations as follows for subsequent analyses: Jeju residents (JEJ), consisting of HAE and JEU, and Korean mainland individuals (KOR), comprising KOA, KMJ, and SEO. We chose to restrict our analysis to these three populations rather than using all global populations used in the demographic analyses to avoid identifying as outliers SNPs that are fixed in 1000 genomes and unique to the Korean populations, an artifact of using many outgroup populations instead of restricting the number of comparison populations to two.

We considered the results from *K* = 3 for downstream analysis as this is the number of ancestry components for which each of the three populations (Jeju residents, mainland Koreans, and Han Chinese) obtain their own distinct component. We considered mainland Koreans and Han Chinese from the South (CHS) as comparison populations for the selection scan. To estimate admixture proportions, we sampled 3% of sites to use as input for Ohana.^34^ We ran qpas, varying *K* from 2 to 3, with a maximum of 450 iterations, and used 0.08 as the minimum likelihood delta between iterations. We used nemeco from Ohana to model the allele frequencies across ancestral components and obtain a matrix of the variances and covariances between them. We then used this matrix to estimate a tree of the ancestry components.

We employed the selscan program in Ohana^34^ to identify SNPs that exhibited significant allele frequency changes in Jeju residents compared to that expected by the genome-wide covariance structure, using a likelihood ratio test. In selscan, this is done by first estimating a covariance matrix of allele frequencies among populations for the genome-wide data. For the specific SNP tested, a scalar variable is then multiplied on the variance component associated with population of interest (in this case the Jeju population), and the value of this scalar parameter is estimated using maximum likelihood. This leads to the establishment of a likelihood ratio test comparing a model in which the allele frequency change is as expected from the genome-wise pattern and one which allows increased allele frequency change for the SNP being tested. In our case, large likelihood ratios indicate support for the hypothesis of increased change in allele frequency in a SNP the Jeju population compared to that expected from the genome-wise pattern.

We used the same 5,673,091 SNPs as in the previous analyses, but eliminated apparent fixed differences between the Korean1K data and the 1000 Genomes data set, assuming that these SNPs were suffering from allele flipping errors (see e.g.,^80^). We verified that a selection of these SNPs were allele flips in the 1000 Genomes project by inspecting the original reads from this project using samtools pileup. A total of 40 SNPs were excluded for this reason, leaving a total of 5,673,051 (See Table S3 for excluded SNPs).

To select outliers from selscan, we identified SNPs with likelihood ratios in the 99th percentile. We kept only outliers with at least one other SNP within in a 200 kb window (+−100kb from the SNP) that also was in the 99th percentile. To select the top SNP from each selection signal, we removed the outliers with a neighboring SNP in a 200kb window (+−100kb of the outlier) that had a higher likelihood ratio. Among our top candidate SNPs, multiple hits were located within a wide peak in chromosome 8. This peak was located around the centromere of chromosome 8 and spanned more than 5Mb, much larger than the 200kb maximum peak size assumed by our filters. Despite multiple apparent hits, the SNPs in this region represent a single, wide peak caused by lower levels of recombination at the centromere of metacentric (and submetacentric) chromosomes^81^ See Figure 6. We therefore kept only the top SNP within that region as the representative SNP of the signal from that peak, the strongest signal of selection in our results. We identified the next 9 strongest signals of selection not found within the peak for a total of 10 SNPs. We performed gene annotation using the GRCh38 annotation (NCBI Accession: PRJNA31257 ID: 3125). We filtered the gff file to extract the gene coordinates and annotated a SNP if it fell within the gene, including intronic regions.

In order to calibrate the p-values obtained from our *selscan*, we used an inflation factor of λ=1.94. The likelihood ratio test *selscan* employs is bounded at zero, implying that the expected distribution of the log-likelihood ratio (LLR) statistic (−2 * LLR) is a 50:50 mixture of a point mass at zero and a $\chi_{1}^{2}$ distribution, i.e. we would expect 50% of the p-values to be equal to one under the null hypothesis. For the genomic control, we therefore excluded the lowest half of all LLRs, all of which had a value of zero. Worth noting is that, in fact,73.9% of all SNPs we examined (4,190,414 out of 5,675,051) had a likelihood ratio of zero. This additional zero inflation is due to the discrete nature of the SNP allele frequency data, and we do not do further correction for this. For the remaining 50% of SNPs (2,836,525 SNPs), we compared their LLRs to the expected $\chi_{1}^{2}$ distribution to determine the inflation factor. Commonly, the inflation factor, λ, is calculated using ratio of the medians of the empirical and theoretical distributions. However, due to the excess of zero values even after excluding the lowest 50%, it would not be appropriate to use the median for genomic control. Instead, we use 99th percentile to correct, i.e. we calculated the inflation factor as

$$\lambda =\frac{P_{99}^{\text{observed}}}{P_{99}^{\chi^{2}}}$$

We divided the LLRs by this inflation factor and recalculated the p-values to obtain the calibrated p-values (Figure S4).

##### Genetic association

We ran an association test using PLINK^44^ which performed a linear regression analysis on the top 10 SNPs from the selection scan and untransformed maximum diastolic blood pressure (BP) during the first simulated dive. The analysis included the following covariates: age; height; weight; dive time; and the first 10 principal components (PCs), to control for any underlying population structure. The linear regression model was adjusted for these covariates, and we used an additive genetic model for SNP analysis. We checked for inflation in the association values with a qq-plot genome-wide and found that values were not inflated (λ = 1.01, Figure S5), suggesting that the first 10PCS properly control for underlying population structure. We therefore continued with association testing using the untransformed maximum diastolic pressure values. Only one SNP from the selection scan, the 4^th^ strongest signal, was significantly associated with maximum diving diastolic BP at the Bonferroni-corrected significance level (0.005) using an additive model: rs66930627 (*p* = 0.0021). When we included age^2^ as an additional covariate to account for non-linear effects, as is common in BP GWAS,^45^ the association remained significant (*p* = 0.0014). We also tested the association within only Jeju participants, to assess whether the association reflects population-specific allele frequency differences between Jeju and Seoul and found that the association remains significant (*p* = 0.0039). We note that we were unable to include use of anti-hypertensives as an additional covariate given that these data were not collected at the time of the study.

The associated SNP, rs66930627, is found in an intergenic region, however it has a FORGEdb score of 7 indicating it likely has a regulatory function. FORGEdb scores range between 0 and 10, with 10 indicating highest likelihood of affecting regulation, and are calculated combining multiple sources of information regarding regulation, including data for transcription factor (TF) binding and chromatin accessibility. Specifically, we used the GTEx portal^82^ to identify a strong expression quantitative trait locus (eQTL) with this SNP and the gene that encodes Fcγ receptor IIA (FcγRIIA, *p* = 0.00012) in skeletal muscle (Data Source: GTEx Analysis Release V8 (dbGaP Accession phs000424.v8.p2). FcγRIIA is a low-affinity Fcγ receptor that binds IgG2 and may influence hypertension through its effect on the pro-inflammatory activities of vascular smooth muscle cells (VSMCs).^46^ Another low-affinity FcγR (FcγRIIB) has been demonstrated to correlate with hypertension directly,^48^ and has been suggested that it influences vascular contraction. Fcγ receptors, therefore, may play a crucial role in regulating diastolic blood pressure via their effect on vasculature. Using GTEx, we also found a strong eQTL with rs66930627 and the pseudogene or lncRNA Heat shock 70kDa protein 7 (HSPA7) (*p* = 0.0000012) in whole blood. Knockdown of HSPA7 has been shown to inhibit the expression of inflammatory mediators, thus highlighting its potential role in vascular inflammation.

##### Selection validation

In order to validate that natural selection has acted on the variant we identified as under selection (using the *selscan* genome-wide selection scan) and associated with our phenotype of interest, we used an alternative strategy for detecting selection. Specifically, we tested selection over time using CLUES2.^35^ Using our previous results from Relate (see demographic history and population divergence), we extracted the subtrees corresponding to the JEJ population using Relate’s SubTreesForSubpopulation feature. We then used the SampleBranchLengths function with the inferred historic JEJ population size to extract 200 samples of the gene tree at the SNP rs66930627 in Newick format. These samples were then converted to the CLUES2 input format. We then ran CLUES2 with the default 450 discretization points and a time cutoff of 450 generations (12150 years) on our sample of trees for 3 distinct models: constant selection through time, a selection breakpoint of 45 generations (1215 years), and a selection breakpoint of 259 generations (6993 years). These generation-to-year conversions are based on using a generation time of 27 years.

##### All of Us association validation

We were also able to validate the association between diastolic blood pressure in All of Us (*p* = 0.040) with the following filters: WGS data, “Are you currently prescribed medications and/or receiving treatment for high blood pressure (hypertension)?” (no, to reduce the confounding effect of antihypertensives) Ages 55–75 (to match the ages in the Korean cohort), and Race: White (to produce an approximately single-ancestry cohort: European). This represented the largest single-ancestry cohort currently available through All of Us with the aforementioned filters. We also removed samples flagged as related, resulting in a final cohort size of *n* = 1376 individuals. We used a multiple linear regression to calculate association using the first 10 PCS, age, height, and weight as covariates, as in the Korean cohort (Table S4). Systolic blood pressure was not significantly associated at the same locus.

### ADDITIONAL RESOURCES

Newly generated, de-identified whole genome sequencing data have been deposited at the National Center for Biotechnology Information (NCBI) Sequence Read Archive (SRA) as PRJNA1235291. They are publicly available as of the date of publication. All original code has been deposited at https://github.com/aguilar-gomez/haenyeo and is publicly available as of the date of publication.

## Supplementary Material

1

## Figures and Tables

**Table 1.** Population divergence quantified by F_ST_ values of relevant populations

POP1	POP2	Weir and Cockerman FST
CHB	CHS	0.00096
JEJ	KOR	0.0034
HAE	JEU	0.00031
CHB	KOR	0.0026
JPT	KOR	0.0030
CHS	KOR	0.0046
JPT	JEJ	0.0047

Jeju resident populations, Haenyeo and Jeju controls (HAE and JEU, respectively), are not meaningfully genetically diverged. Mainland Koreans (KOR) appear more diverged from Jeju residents (JEJ) than from northern Han Chinese (CHB) and Japanese (JPT); however, this may reflect low effective population size (*N*_*e*_) in Jeju relative to CHB and JPT rather than true differences in divergence. Both Korean populations, Jeju and mainland, had relatively low divergence with Japanese and Chinese populations.

**Table:** KEY RESOURCES TABLE

REAGENT or RESOURCE	SOURCE	IDENTIFIER
Deposited data		
Haenyeo (HAE), Jeju (JEU), Seoul (SEO) genomes	This paper	SRA: PRJNA1235291
Korean Genome Project: Korean1K (KOA) genomes	Jeon et al.^33^	https://www.science.org/doi/10.1126/sciadv.aaz7835
1000 Genomes Project: Japanese in Tokyo (JPT), Han Chinese in Beijing (CHB), Southern Chinese population (CHS), Kinh Vietnamese (KHV), British in England and Scotland (GBR) and Yoruba from Nigeria (YRI)	Genomes Project et al.^65^	http://ftp.1000genomes.ebi.ac.uk/vol1/ftp/data_collections/1000_genomes_project/release/20181203_biallelic_SNV/
All of Us Genomes	All of Us Research Program	https://www.researchallofus.org/
Software and algorithms		
FastQC v0.11.8	Andrews^66^	http://www.bioinformatics.babraham.ac.uk/projects/fastqc/
PRINSEQ-lite 0.20.4	Schmieder and Edwards^67^	https://prinseq.sourceforge.net/
bwa-mem	Li^68^	https://github.com/lh3/bwa
samtools	Li et al.^69^	https://www.htslib.org/
GLIMPSE v1.1.1	Rubinacci et al.^32^	https://odelaneau.github.io/GLIMPSE/glimpse1/
bcftools	Danecek and McCarthy^70^	https://github.com/samtools/bcftools
ngsToolsV2	Hanghoj et al.^71^	https://github.com/mfumagalli/ngsTools
plink1.9	Purcell et al.^44^	https://www.cog-genomics.org/plink/1.9/
plink2	Chang et al.^72,73^	https://www.cog-genomics.org/plink/2.0/
Ohana	Cheng et al.^34^	https://github.com/jade-cheng/ohana
Treemix	Pickrell and Pritchard^74^	https://bitbucket.org/nygcresearch/treemix/wiki/Home
Admixture Bayes	Nielsen et al.^75^	https://github.com/avaughn271/AdmixtureBayes
Relate	Speidel et al.^76,77^	https://myersgroup.github.io/relate/
CLUES2	Vaughn and Nielsen^35^	https://github.com/avaughn271/CLUES2
pong	Behr et al.^78^	https://github.com/ramachandran-lab/pong
custom scripts and whole pipeline	This paper	https://github.com/aguilar-gomez/haenyeo/

## Highlights

- Evidence of selection that may increase the safety of diving during pregnancy
- Regular diving increases the magnitude of bradycardia in response to dive stimulus
- The Haenyeo may represent the second known population evolved for diving
