# Supplementary material for: Genetic and training adaptations in the Haenyeo divers of Jeju, Korea
Source: Cell Rep. Author manuscript; Available in PMC 2025 Jun 3. (PMC12129667; doi:10.1016/j.celrep.2025.115577)
Supplement: 1 [file NIHMS2074498-supplement-1.pdf]

Cell Reports, Volume [redacted]

Supplemental information

Genetic and training adaptations

in the Haenyeo divers of Jeju, Korea

Diana Aguilar-Gómez, Jacob Bejder, Jonathan Graae, Yelin Ko, Andrew Vaughn, Kendell Clement, Martin Tristani-Firouzi, Joo-Young Lee, Nikolai B. Nordsborg, Rasmus Nielsen, and Melissa Ilardo

SUPPLEMENTAL FIGURES

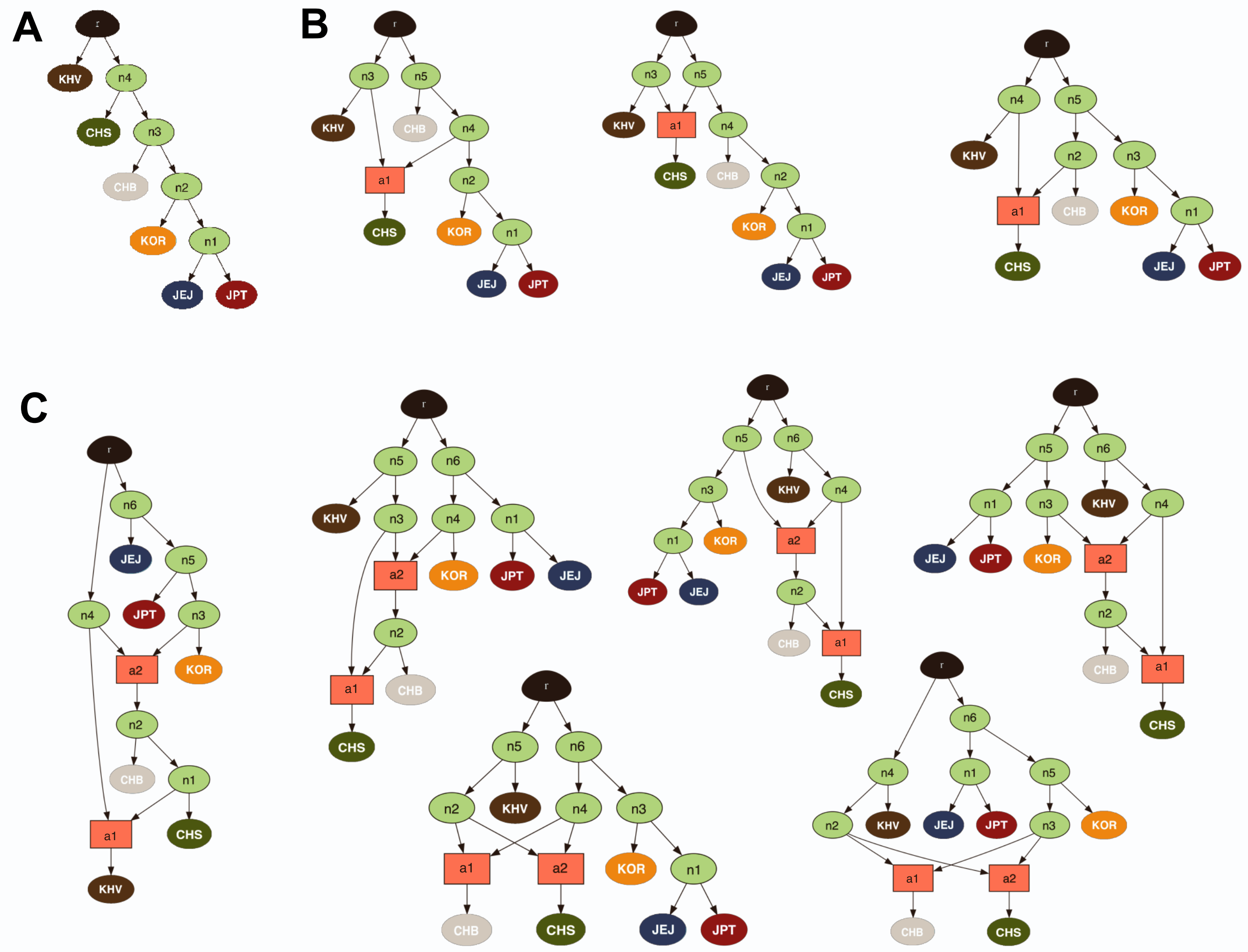

Supplemental Figure 1. Population history trees show possible historical relationships between Han Chinese in Beijing (CHB), Han Chinese in the south (CHS), Japanese (JPT), mainland Koreans (KOR), and Jeju Islanders (JEJ). A) AdmixtureBayes results when allowing 0 admixture events. 100% of all sampled graphs have this topology. B) Results when allowing 1 admixture event. Posterior probabilities of topologies are (from left to right) 87.8%, 10.6%, and 1.6%. C) Results when allowing 2 admixture events. Posterior probabilities of all topologies shown are at least 10%.

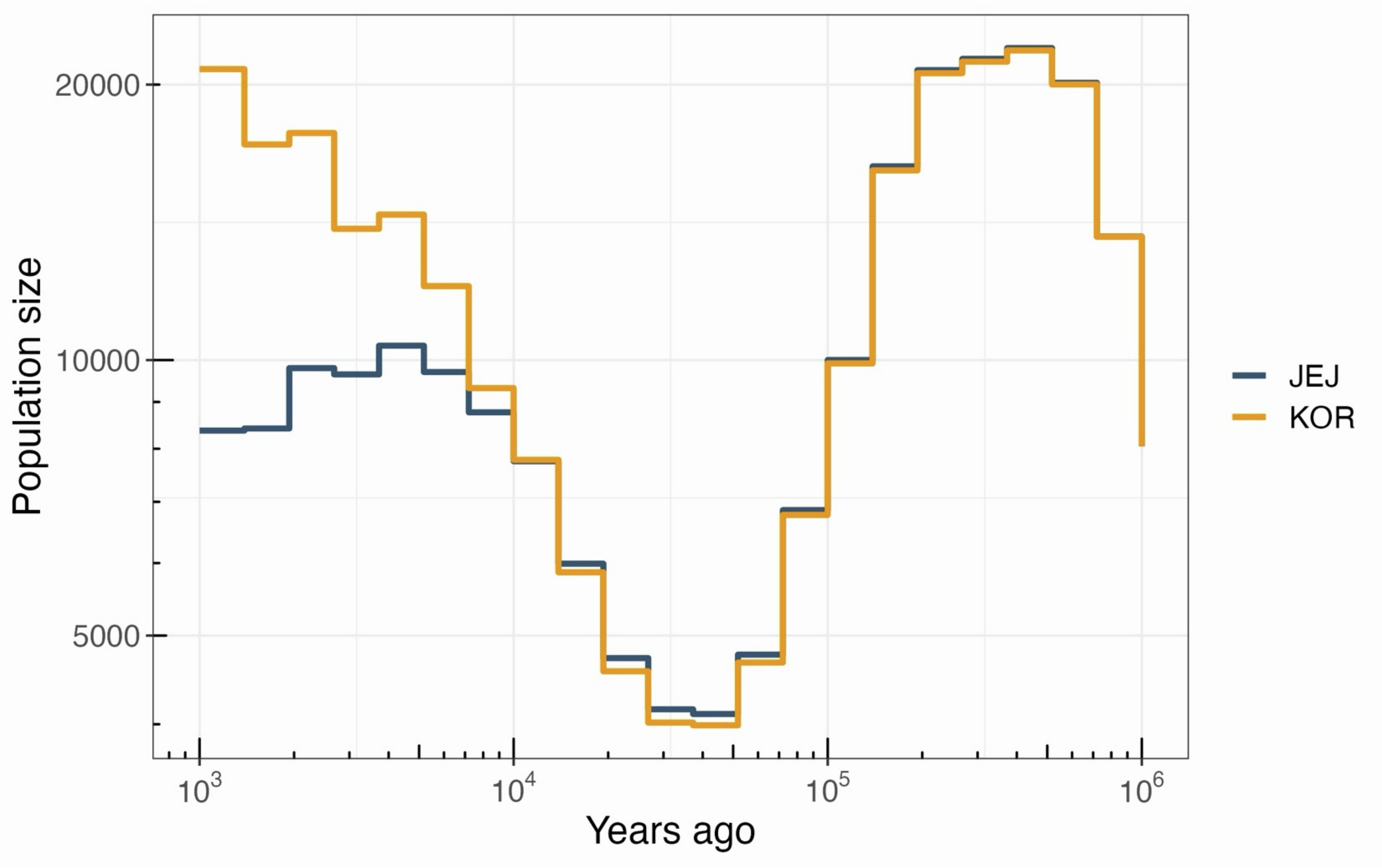

Supplemental Figure 2. Population-specific estimates of population size for Jeju (JEJ) and mainland Korea (KOR) using genome-wide genealogies. The subpopulation histories share a trajectory prior to 10,000 years ago, at which point they begin to diverge. From these analyses, we estimate the split between Jeju and mainland Korea occurred between 5,000 and 7,000 years ago. Jeju appears to have suffered a bottleneck roughly 1,200-3,000 years ago.

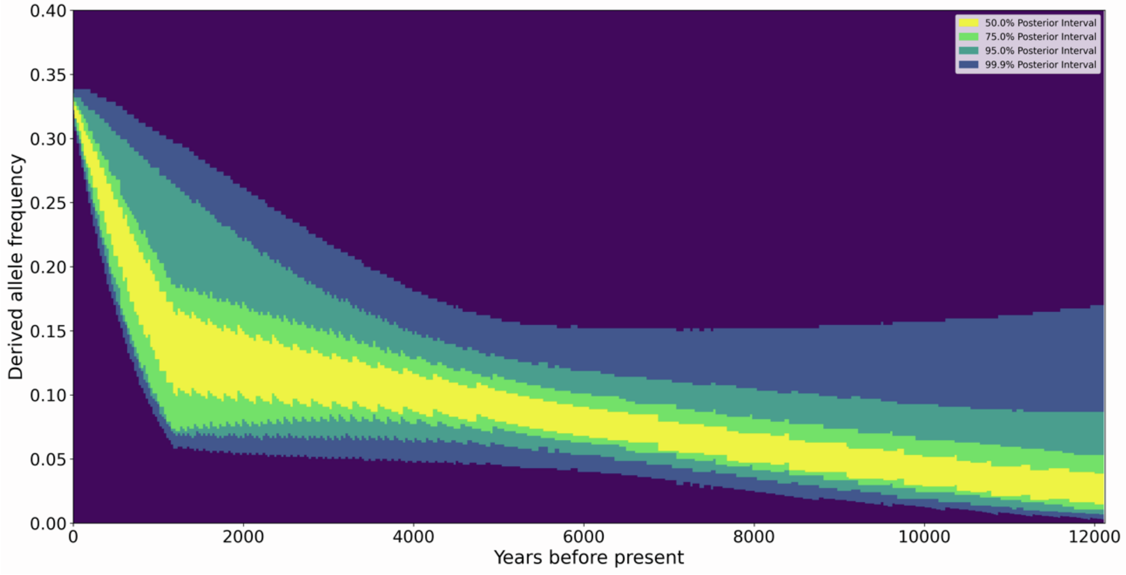

Supplemental Figure 3. Reconstructed historic allele trajectory of the derived C allele at the SNP rs66930627 in the JEJ population using CLUES2. This trajectory uses the 2-epoch model with a selection coefficient change point at 1200 years ago. $\hat{s}^{MLE} = 0.021$ in the more recent time period and $\hat{s}^{MLE} = 0.004$ in the older time period.

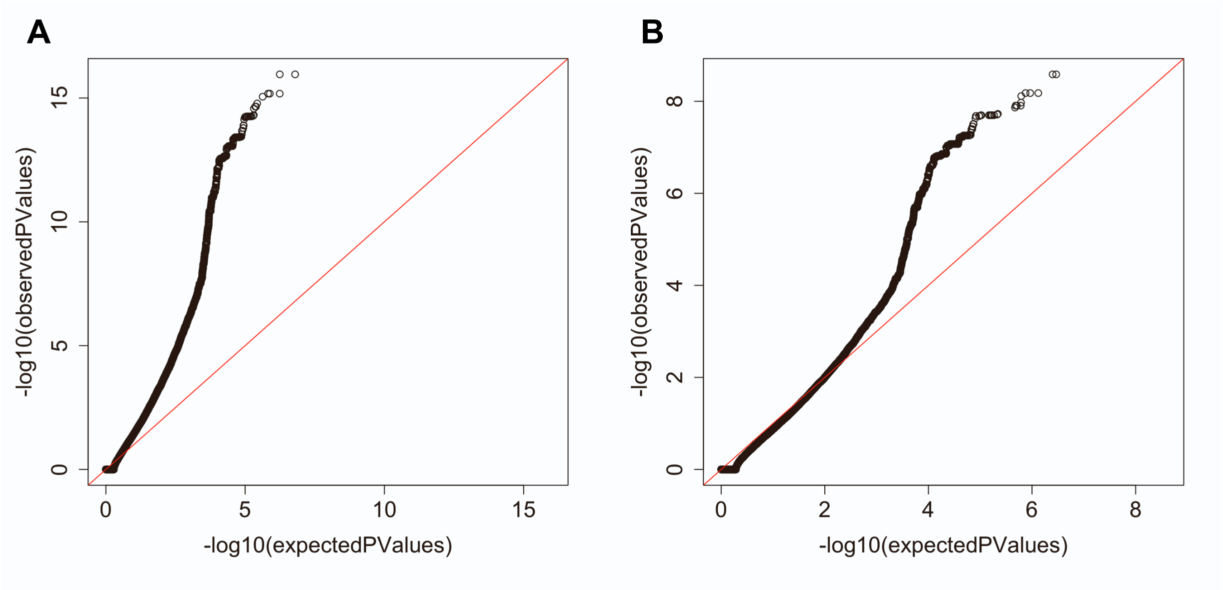

Supplemental Figure 4. Two qq-plots representing (A) the untransformed p-values and (B) the inflation corrected p-values resulting from the genome-wide selection scan results (λ = 1.94).

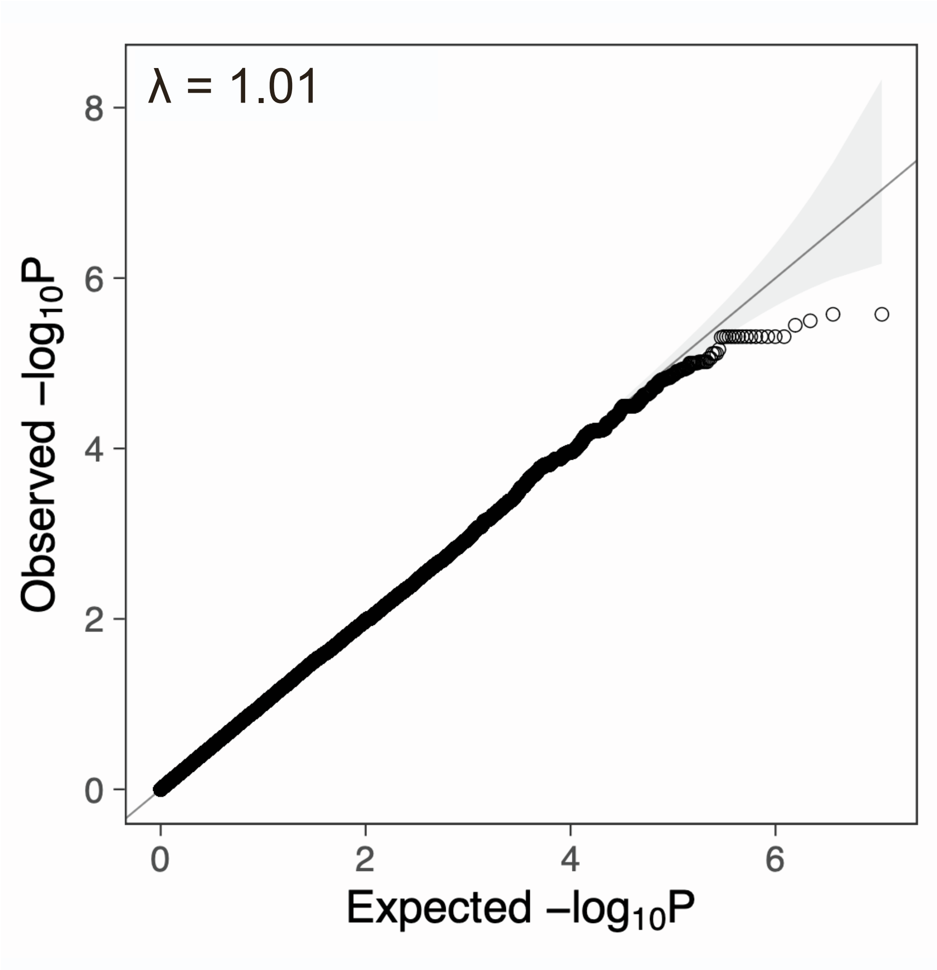

Supplemental Figure 5. A qq-plot for untransformed diastolic blood pressure association p-values genome wide demonstrates an absence of inflation in these values (λ = 1.01).

SUPPLEMENTAL TABLES

**Original Population Label**	**New Population Label**	**CHS Ancestry**	**JEJ Ancestry**	**KOR Ancestry**
Korean1K	Jeju	0.000001	0.999998	0.000001
Jeju	Korean Mainlander in Jeju	0.087736	0.117498	0.794766
Jeju	Korean Mainlander in Jeju	0.000001	0.137829	0.862170
Jeju	Korean Mainlander in Jeju	0.000001	0.108534	0.891465
Jeju	Korean Mainlander in Jeju	0.082885	0.000001	0.917114
Jeju	Korean Mainlander in Jeju	0.017095	0.053652	0.929253

Supplemental Table 1 **Individuals re-labeled according to genetic ancestry based on admixture ancestry proportions.** Several individuals were re-labeled for downstream analyses to better align with their ancestral proportions. We re-labelled an individual if more than 75% of their ancestry was derived from a different population than that to which they were originally assigned.

Chromosome	position	selection p-value	gene name	rsid	association p-value
chr8	43499825	$2.60x10^{-9}$	LOC105379397	rs118132530	0.3641
chr3	53824959	$5.46x10^{-8}$	CHDH	rs62250931	0.4514
chr6	44215128	$1.80x10^{-7}$	POLR1C, MYMX	rs147937252	0.1857
chr1	161704797	$2.87x10^{-7}$	x	rs66930627	$2.1x10^{-3}$
chr8	14488480	$5.35x10^{-7}$	SGCZ	rs141632375	0.2974
chr4	12993585	$9.96x10^{-7}$	x	rs74627435	0.4072
chr5	139659720	$1.05x10^{-6}$	CXXC5	rs549239706	0.3361
chr7	25883045	$1.20x10^{-6}$	x	rs139022115	0.7378
chr7	96446404	$1.29x10^{-6}$	x	rs73239797	0.5894
chr6	153387842	$1.33x10^{-6}$	LOC105378066	rs74374474	0.1965

Supplemental Table 2 **Top 10 strongest signals of selection in the Jeju genome**. Results of the Ohana selection scan identify the top 10 SNPs found in regions under selection in the Jeju population as well as the p-value of their association with diving diastolic BP. Only one SNP, rs66930627, was found to be both naturally selected in Jeju and associated with diastolic BP.

chr1:145928008	chr2:241728674	chr6:25708794	chr10:76514820	chr13:106662576
chr1:145998776	chr2:79867345	chr6:37299180	chr11:54588661	chr14:75897187
chr1:158208165	chr3:174321193	chr7:53214456	chr11:54830077	chr15:94923307
chr1:206116320	chr3:17818282	chr8:119343459	chr12:115244731	chr18:69381410
chr1:206116501	chr4:51963971	chr8:72341591	chr12:13476447	chr18:72482932
chr1:7501170	chr5:115184519	chr9:86285193	chr12:5664803	chr18:77078804
chr1:83891838	chr5:178932686	chr10:47149245	chr12:71566221	chr21:17429885
chr2:154891113	chr5:30119027	chr10:47298786	chr12:76857943	chr22:16673074

Supplemental Table 3 **List of 40 SNPs excluded from selection analyses due to allele flipping.**

Genotype	n	mean diastolic BP
C/C	562	80.52
C/T	613	81.62
T/T	201	82.02

Supplemental Table 4. **All of Us cohort diastolic BP stratified by genotype**. Each added T allele at rs66930627 corresponded to on average an increase in diastolic BP of 0.75mmHg in the All of Us cohort.
